# Research trends in mobile payment adoption: Research trends and agenda

**DOI:** 10.12688/f1000research.159551.1

**Published:** 2025-03-31

**Authors:** Alejandro Valencia-Arias, Jesus Alberto Jimenez Garcia, Gustavo Moreno-López, Aaron José Alberto Oré León, Lucia Palacios-Moya, Jackeline Valencia, Martha Benjumea-Arias

**Affiliations:** 1 School of Industrial Engineering, Universidad Senor de Sipan, Chiclayo, Lambayeque, 14001, Peru; 2 Dirección de Planificación y Desarrollo Institucional, Universidad Senor de Sipan, Chiclayo, Lambayeque, 14001, Peru; 3 Rectoría, Institucion Universitaria Marco Fidel Suarez, Medellín, Antioquia, 50030, Colombia; 4 Instituto de Investigación y Estudios de la Mujer, Universidad Ricardo Palma, Santiago de Surco, Lima, Peru; 5 Escuela de posgrado, Universidad Senor de Sipan, Chiclayo, Lambayeque, Peru; 6 Facultad de Ciencias Económicas y Administrativas, Instituto Tecnologico Metropolitano, Medellín, Antioquia, 050034, Colombia

**Keywords:** Mobile banking, electronic payments, Digital payments, Technology acceptance, PRISMA.

## Abstract

This study explores the adoption of mobile payments as a research field to understand the technological, socioeconomic, and cultural factors that influence the understanding of the users’ experience and satisfaction and attract more users. It is vital to examine the research trends in the adoption of mobile payments from a systematic literature review using PRISMA 2020 to select 63 documents from Scopus and WOS. Among the results, the most used models for this analysis are extended TAM and extended UTAUT. The most representative variables in the studies are social influence, security perception, risk perception, trust and perceived usefulness. It is concluded that the future research agenda should focus on topics such as biometric authentication, payment flexibility and contactless payments. In addition, from the business perspective, there is a focus on designing innovative interfaces that are more intuitive for users.

## Introduction

In recent years, digitization has permeated an increasing number of aspects of the current social configuration, making it possible to prominently position tools such as mobile payments, which have been increasingly positioned as a convenient and safe way to carry out financial transactions. In this sense, authors such as Bojjagani et al. (2023) explain that mobile payments refer to the use of mobile devices, such as smartphones or tablets, to carry out financial transactions, including money transfers, online purchases and payments or bill payments, which has allowed us to observe an increasingly greater increase in scientific production around the understanding of the factors that influence the adoption of this technology.

In this sense, one of the main discussion factors around the adoption of mobile payments has been the initial trust that influences both the understanding of adoption and the intention to continue using mobile payments, according to authors such as Talwar et al. (2020). In addition, this technology has been largely prevalent in the pandemic and post pandemic period due to COVID-19, which is why frameworks have been proposed that integrate the main models that allow increasing the levels of knowledge around the adoption of this technology to this specific historical moment, as well as other more specific variants that have unified models to understand the adoption of disruptive technologies in the service sector ( Schmidthuber et al., 2020), or in specific contexts, such as Gupta and Arora (2020), who analyzed the intention of Indian consumers to adopt mobile payment systems.

Other authors, such as Kalinić et al. (2020), have shown that the understanding of the factors that affect the adoption of mobile payments not only is conditioned by factors of technology but, on the contrary, is essential to understand variables associated with socioeconomic and cultural aspects when studying the moderating impact of gender on the acceptance of mobile payment systems, finding that men report a greater probability of adopting these systems than women.

The adoption of mobile payments has been highly influenced in recent years by smartphones’ growing role in the lives of consumers, which is consistent with the increase in demand for digital financial services. In this sense, companies have begun to invest increasingly in the development of mobile payment platforms in such a way that they can take advantage of all the opportunities offered by this type of payment system. In addition, the COVID-19 pandemic has accelerated the transition to digital payments, which has generated greater interest in this topic.

Based on the above, other authors have highlighted the current importance of recognizing and exploring all the challenges and opportunities derived from the growth of the adoption of mobile payment platforms from multiple perspectives, such as business models ( Jocevski et al., 2020), awakening in organizations a recognition of the importance of specific strategies and solutions, such as mobile wallets, mentioned by Mombeuil (2020), which have been important for all those companies that seek to improve elements associated with the user experience and, with this, significantly increase their customer or consumer base. Likewise, in business terms, authors such as Verma et al. (2020) speak of the importance of considering all the elements associated with government regulations so that organizations can focus on the aspects properly associated with the adoption or intention to continue using this type of service.

In terms of the adoption of mobile payments, it is essential to analyze the behavior of use of this type of system in specific populations, not only from the geographical point of view but also from the demographic point of view, as evidenced in ( Wei et al., 2021), where organizations speak of the importance of understanding these factors from the young generation, which has been complemented by Karimi and Liu (2020), which, in addition, expand on the need to study the differential impact of the mood of this type of consumer, as well as that mentioned by Teng and Khong (2021) on the opportunity to take advantage of emerging technologies such as Big Data, which provide fundamental information in terms of consumer segmentation, understanding their diversity in the use of this type of system.

However, although the adoption of mobile payments is positioned as a relevant issue and, in that sense, it is currently booming as a result of the development of new mobile technologies, there are still several important research gaps due to the lack of complete review studies that unify the current state of knowledge of the subject, as well as its specificities, such as the understanding of some psychobehavioural or cultural elements ( Wasil et al., 2020), the incidence of consumer behavior in terms of adoption of mobile payments ( Flavian et al., 2020), or how the adoption of these systems occurs in the post pandemic context in other specific societies ( Rafdinal & Senalasari, 2021). Therefore, it is necessary to carry out a systematic review of the literature (SRL) to identify the primary studies and their characteristics and therefore to guide future research agendas. In this sense, this article aims to explore the scientific literature on mobile payment adoption in order to guide future research. To achieve this, the following broad and specific research objectives are proposed:

**RO1:** Identify and analyze bibliometric trends in the adoption of mobile payments.

**RO2:** Investigate the evolution of the main keywords for analysis in the scientific literature on the adoption of mobile payments.

**RO3:** Evaluate the theories used by researchers to determine the adoption of mobile payments.

**RO4:** Analyze the main variables used to understand the adoption of mobile payments.

**RO5:** Identify research gaps in the literature on mobile payment adoption and propose future research questions based on these gaps.

**RO6:** Design a research agenda that integrates identified gaps and growing and emerging research topics on mobile payments adoption.

This article consists of an introductory section, where the definition, importance and problems associated with the adoption of mobile payments are analyzed. The methodological section follows, where the parameters through which the systematic literature review will be developed are defined, as well as the results associated with the research and discussion of these, where research gaps are raised, and the design of a research agenda. Finally, a conclusion on the main aspects is presented.

## Methods

Once the research objective has been identified, an exploratory search is designed that proposes a bibliometric analysis, which facilitates the evaluation of the scientific publications found in the literature. The bibliometric analysis favors the discovery of emerging trends in the production of the authors, the journals, as well as collaboration patterns and research components ( Donthu et al., 2021). Additionally, to enhance the elements of detail and replication, a bibliographic review process was carried out, taking as a reference the international PRISMA 2020 statement, which defines the eligibility criteria, sources of information, search strategy and data management to be executed on the selected data sources, as evidenced in Page et al. (2021) and specified in Valencia-Arias et al. (2023).

### Inclusion and exclusion criteria

For the present research, the eligibility criteria used for the selection of articles are divided into inclusion and exclusion criteria based on the international PRISMA 2020 statement, being that in the case of the inclusion criteria of this research on the adoption of mobile payments, concepts such as adoption, technology, mobile and payment are found in both the title and keywords of the publications to be analyzed, thus guaranteeing that the publications found are relevant for this research.

For the exclusion criteria, three consecutive complex selection phases were defined based on the international PRISMA 2020 statement. In the first phase, documents with incorrect indexing are excluded, starting from reading the titles of the selected documents. For the second phase, those articles whose full text is unavailable are discarded; however, this element does not apply to bibliometric analysis. Since metadata analysis is used, the aforementioned is designed explicitly for systematic literature reviews, where the full text is analyzed. Finally, for the third and last phase, all those documents derived from conference proceedings are eliminated, including those considered irrelevant that may limit the analysis that allows the fulfilment of the objective to be fully achieved.

### Information sources and search strategy

For the search for scientific and academic information related to the acceptance of mobile payments, Web of Science and Scopus were selected. They are the most widely used databases in analysis processes and scientific evaluation because they are multidisciplinary bases that cover many journals and offer resources that are very helpful for re-searchers ( Codina et al., 2020).

For correct execution of the search in the databases, the international PRISMA 2020 statement was considered, which mentions the importance of defining the inclusion criteria and the keywords. For this reason, two different search equations are defined, which change according to the database interface, thus ensuring that all the keywords are found in the documents that yield the search results. As mentioned above, the search equations are as follows:

((TITLE ( “Adoption” OR “acceptance” OR tam OR “Technolog* acceptance model” OR tpb OR “Theory of Planned Behav*”) AND TITLE ((mobile) AND ( pay* OR compensation OR remittance OR remuneration OR deposit))) OR (KEY (“Adoption” OR “acceptance” OR tam OR “Technolog* ac-ceptance model” OR tpb OR “Theory of Planned Behav*”) AND KEY (mobile AND (pay* OR com-pensation OR remittance OR remuneration OR deposit))))

((TI= (“Adoption” OR “acceptance” OR tam OR “Technolog* acceptance model” OR tpb OR “Theory of Planned Behav*”) AND TI= (mobile AND (pay* OR compensation OR remittance OR remuneration OR deposit))) OR (AK= (“Adoption” OR “acceptance” OR tam OR “Technolog* acceptance model” OR tpb OR “Theory of Planned Behav*”) AND AK= (mobile AND (pay* OR compensation OR remittance OR remuneration OR deposit))))

### Data management

Initially, the search yielded 721 results, of which 204 belong to Web of Science and 517 to Scopus. These results range from the first publication in 2004 to the current year, 2023. The articles obtained were processed with the help of Microsoft Excel ^®^ tools and VOS viewer. With the first tool, the data were stored, and the exclusion criteria defined above were applied. Finally, 63 publications were analyzed. In addition, with the help of the second tool, the graphs of bibliometric indicators were generated to facilitate data analysis.

### Selection process

**Figure 1.**
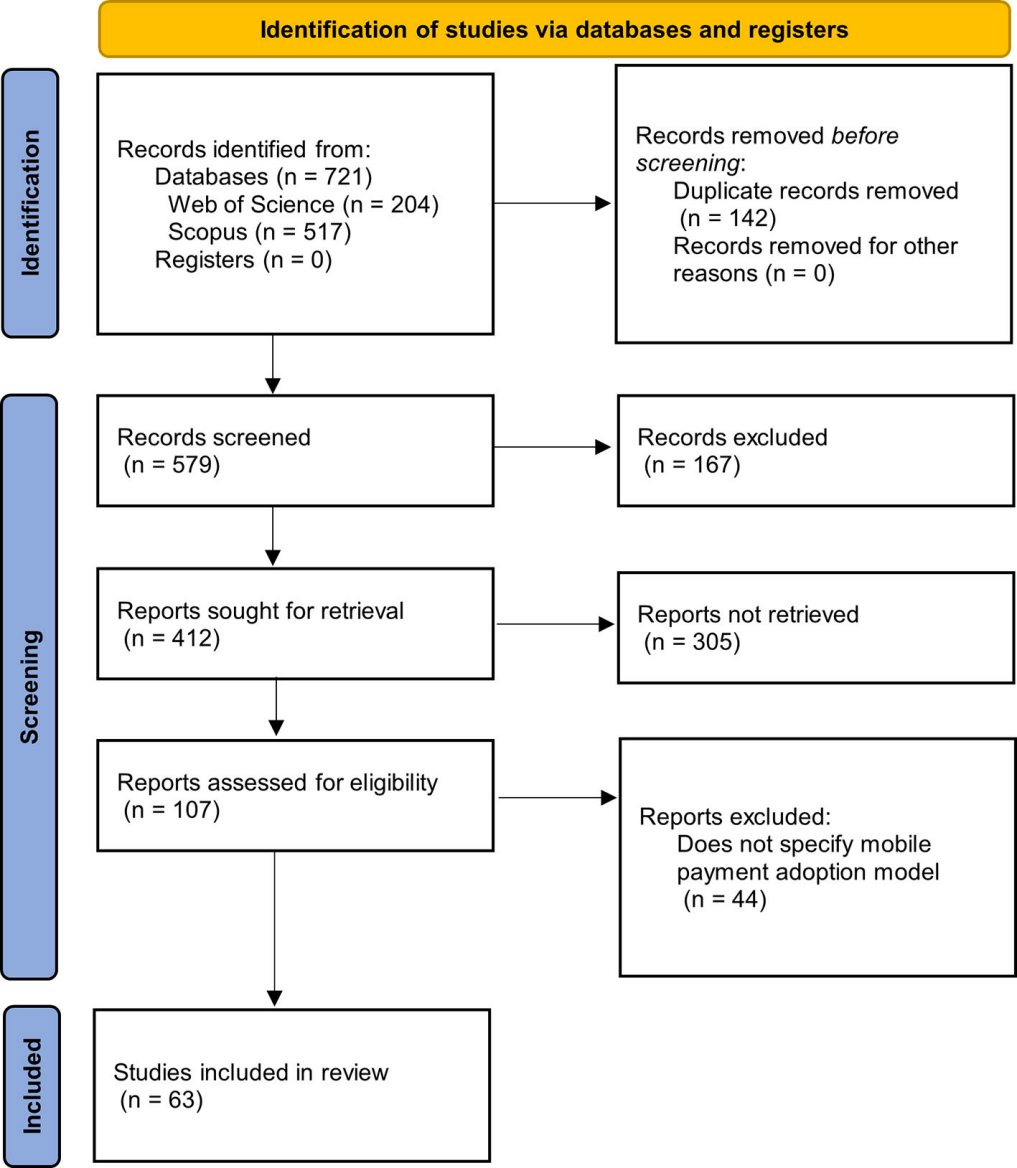
Flowchart for PRISMA systematic literature reviews. **Alt text** **Figure 1**. Flowchart illustrating the PRISMA systematic literature review process.

To reduce the bias of the information found in this research, the articles were independently chosen by the authors, as evidenced by the PRISMA 2020 statement, to conduct an exhaustive review of the selection and analysis of the publications. Additionally, to resolve the differences found in each exclusion process, a detailed analysis was carried out that allowed us to identify similarities or convergences in the selected articles. Finally, Figure 1 summarizes the methodological design used, which is represented using a flow diagram as established by the international PRISMA statement.

The diagram in Figure 1 allows us to follow the step-by-step identification of the articles, starting with the search in each of the databases, where the formulated search equation was used, proceeding with the elimination of duplicate articles, to later continue with the exclusion phases, to finally find the number of articles that will be applied to the metadata analysis.

### Effective bibliometric review

To enhance the quality and methodological transparency of this bibliometric analysis on the adoption of mobile payments, it is recommended to include a reference or detailed guide on how to conduct an effective bibliometric review. This should incorporate examples of relevant studies that illustrate best practices in this field, as demonstrated in Chauhan and Yadav (2024) and Agarwal et al. (2023). This sentence is already well-written and meets the desired characteristics. No changes are necessary.

## Results

This section presents the findings that respond to the objective of exploring the scientific literature on the adoption of mobile payments to guide a research agenda for the development of future research and to the research questions formulated: a) What are the bibliometric trends on the adoption of mobile payments? b) What is the evolution of the main keywords for analysis in the scientific literature on mobile payment adoption? c) What theories are used by researchers to determine the adoption of mobile payments? d) What are the main variables used to understand the adoption of mobile payments? e) What research gaps are identified in the mobile payment adoption literature, and what future research questions can be asked? f ) What elements should a research agenda have that integrate the identified gaps and the growing and emerging research themes on the adoption of mobile payments? This gives way to the estimation of bibliometric indicators for identifying the adoption factors of mobile payments. For this, research trends were analyzed by studying the behavior dynamics of authors, journals and countries, the evolution of the subject, the keywords, and the use of theories and variables that characterize technology adoption from the thematic component.

### Bibliometric aspects of research on mobile payment adoption factors

**Figure 2.**
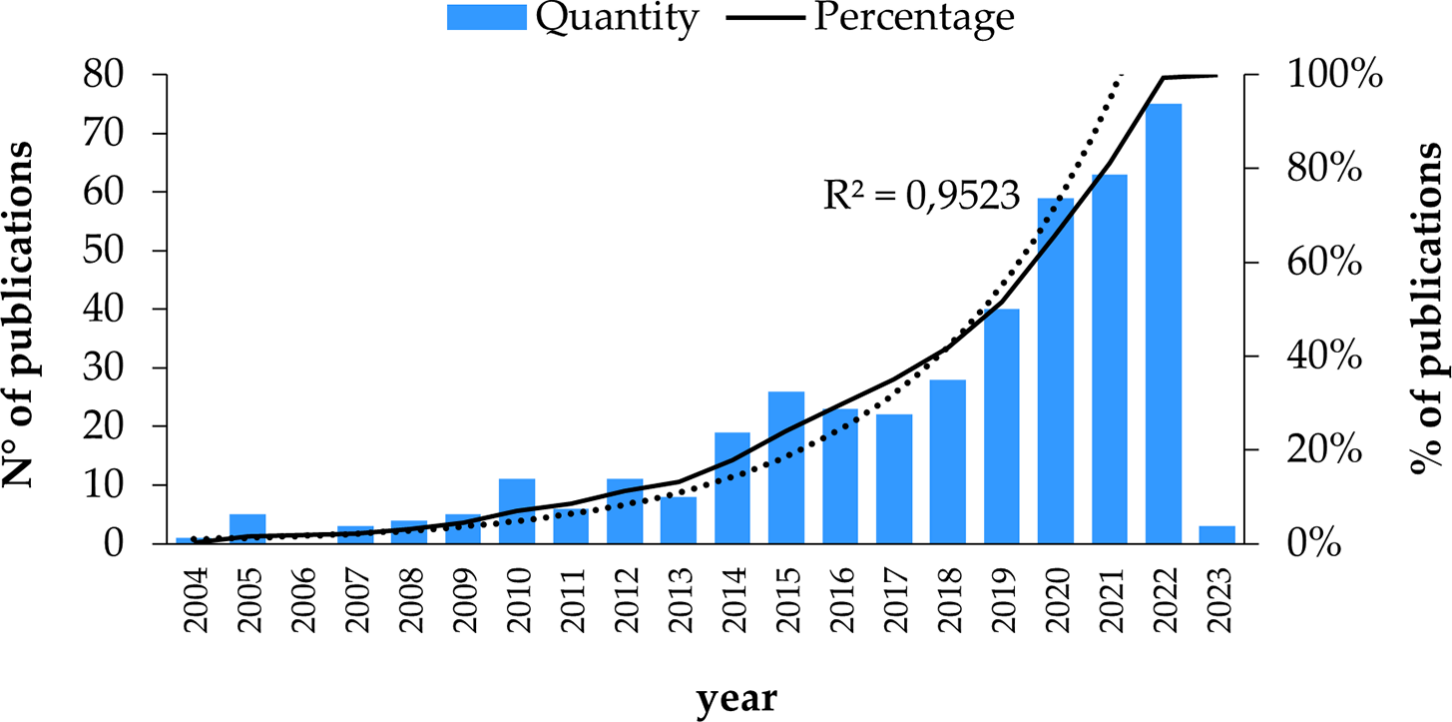
Publications by year. **Alt text** **Figure 2**. Graph depicting the number of publications over the years.

The analysis of these bibliometric indicators was estimated from the 412 studies before eliminating the documents that did not have access to the full text. Exponential growth in publications per year is shown as the years go by, as seen in Figure 2, making evident the use of the subject and the growing interest in adopting mobile payments. These studies range from 2004 to 2023; the most relevant years are 2021 and 2022. The year 2022 has the most publications, with a total of 75, where the objective is to analyze the influence of the dimensions of quality and confirmation on the continuation intentions of users to use electronic wallet applications. These dimensions are usefulness, ease of use and security, which play an important role in the decision-making by users to continue using electronic wallets ( Abbasi et al., 2022).

Subsequently, in 2021, there are 63 publications. The research focuses on the type of mobile payment that uses QR code since the acceptance of mobile payment still has potential for improvement, for which this study seeks to establish the critical antecedents that influence the intention of adopting mobile payments, specifically in the type of mobile payment that uses the QR code technology, through an extended mobile technology acceptance model, this is provided with numerous practical implications, Because the results may not coincide precisely with the adoption of mobile payment with QR code in all countries, as there are significant differences between them. These differences could be either in their culture, ethnicity, or economic development, among others, that probably influence the adoption of this technology ( Yan et al., 2021).

**Figure 3.**
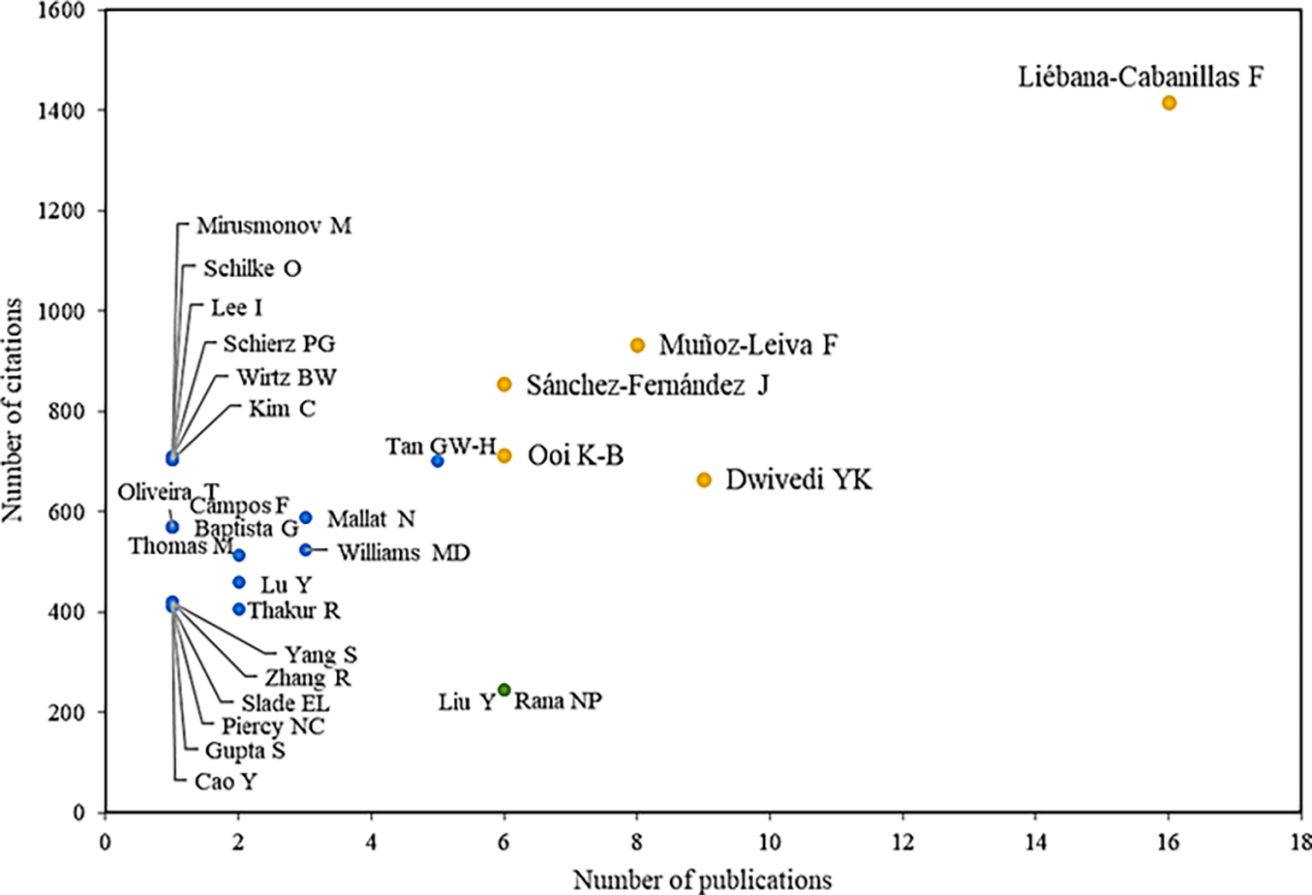
Authors with the highest number of citations and publications. **Alt text** **Figure 3**. Chart showing authors with the highest number of citations and publications.

In Figure 3, the main authors are identified in terms of productivity and impact in the scientific literature on the adoption of mobile payments, evidencing the author Liébana-Cabanillas F as the primary research reference by positioning himself as the author who publishes the most, with 16 articles and, in addition, the most cited, with a total of 1400 citations, and that, meanwhile, accounts for articles that analyze the acceptance of mobile payments by users, accounting for factors such as external influence, ease of use, attitude, usefulness, confidence, risk and age, the latter being a significant factor in terms of adoption (F. J. Liébana-Cabanillas et al., 2014). Likewise, other studies account for the intention to adopt specific systems such as Apple Pay, understanding that today’s society is transitioning to cashless systems (F. J. Liébana-Cabanillas et al., 2020).

Among the main references are also the authors Muñoz-Leiva F., with eight publications and 900 citations associated with their scientific activity. In addition, it is known that these authors have collaborated with Liébana-Cabanillas in one of the main articles in the scientific field, through which an attempt is made to identify the role of the user’s gender in terms of acceptance of mobile payments, concluding that by providing alternatives and segmentation strategies, this new business opportunity can be consolidated under new technological developments (F. J. Liébana-Cabanillas et al., 2014).

On the other hand, there is the author Dwivedi YK with nine publications and 664 citations in his scientific production on the adoption of mobile payments, which is evidenced in his research on how mobile payments would be one of the most successful mobile services in the future. However, his work also shows that until that date, a limited acceptance had been achieved in developed countries, finding that social influence, performance expectation, perceived risk and innovation significantly influenced the intentions to adopt this method ( Slade et al., 2015). On the other hand, he shows how digital payments have great potential for millions of people in developing countries by offering financial services to users who are not banked, showing that the expectation of performance and the perceived useful-ness are key to the behavior of the consumer when using mobile payments and that the perceived risk keeps them from using it ( P. P. Patil et al., 2017).

**Figure 4.**
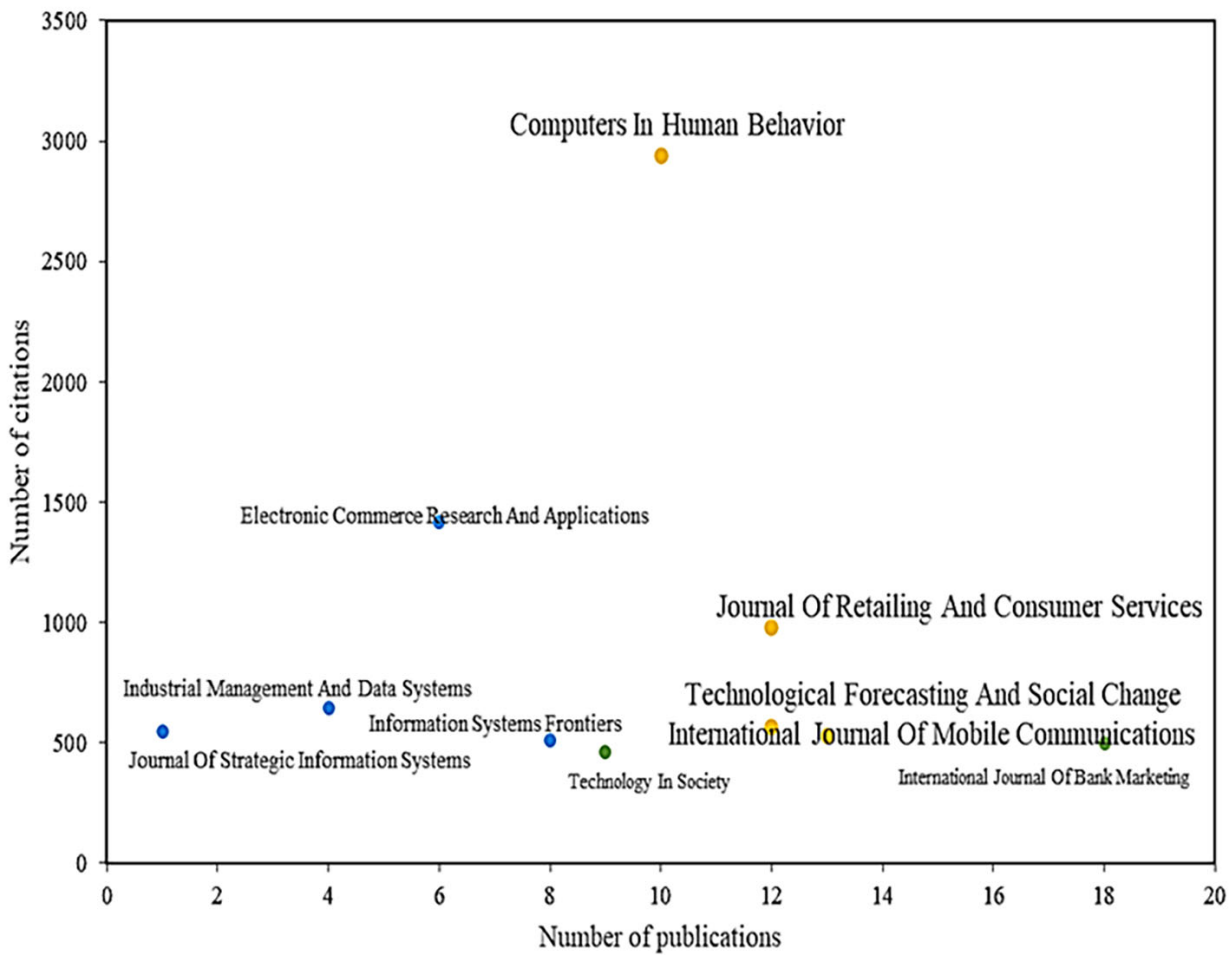
Magazines with the highest number of citations and publications. **Alt text** **Figure 4**. Chart displaying magazines with the highest number of citations and publications.

Likewise, Figure 4 shows the results of the literature review to identify the main scientific journals in terms of dis-semination of articles on the adoption of mobile payments, identifying that the journal Computers in Human Behavior, with a total of 10 publications and more than 2,900 citations, is positioned as the leading magazine in the field. This journal reports articles that study the limitations that exist in the rapid adoption by users of mobile payment services since, despite the advantages offered by this system, adoption has been slow in order for these to be accepted more easily and can continue to prosper, tangible benefits must be provided to the consumer. This can be achieved through links with other projects, such as discounts, loyalty and others that appear along the way ( Johnson et al., 2018).

Another of the main magazines in the scientific field is the Journal Of Retailing And Consumer Services, which has published a total of 12 articles on the subject and with a total of 977 citations, with the main article analyzing the adoption by consumers of proximity mobile payment technology, identifying variables such as perceived value, usefulness, hedonic and social benefits, as well as drivers such as financial and privacy risks that influence adoption ( de Kerviler et al., 2016). Other studies, such as[4], have investigated the role of information and service quality in terms of initial trust in mobile payments and how this affects perceived usefulness and adoption.

Another important journal in the scientific literature is Technological Forecasting And Social Change, which accounts for 13 publications and 527 accumulated citations. In some studies that address the specific characteristics that allow us to understand how technology impacts the intention to use mobile payments, despite the high rate of mobile phones that currently exist and the considerable investments that service providers make in obtaining new technologies, the results of this study reveal that the intention to use these services is seen as highly affected by useful-ness, compatibility, innovation, social media, and perceived social influence but is also affected by perceived risk ( Schmidthuber et al., 2020).

**Figure 5.**
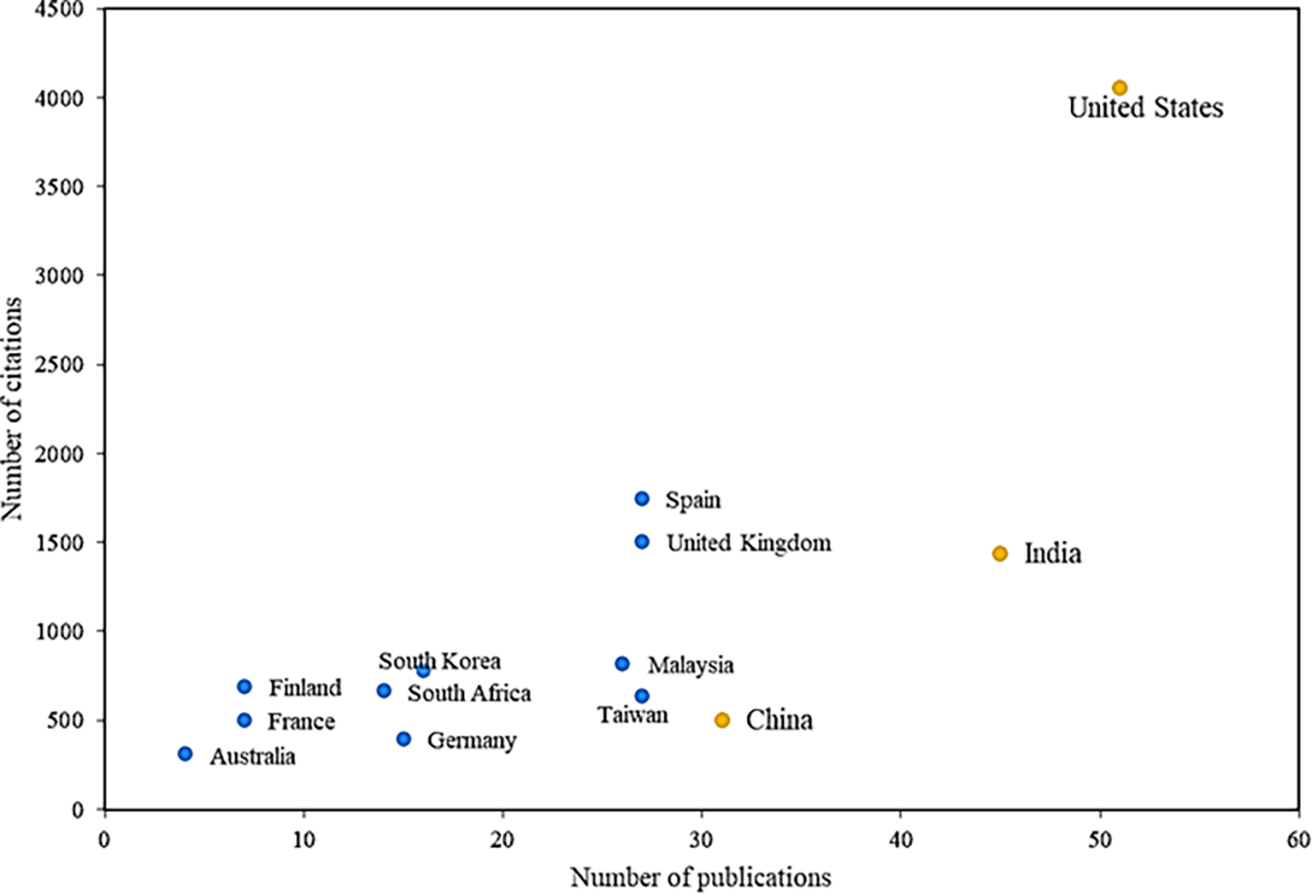
Countries with the highest number of citations and publications. **Alt text** **Figure 5**. Map or chart indicating countries with the highest number of citations and publications.

Figure 5 analyses the most prolific countries with the greatest impact on the subject. In global terms, it is evident that the United States is the leading country in research on the adoption factors of mobile payments, being the most productive, with a total of 51 articles on this research field, having research that focuses on factors such as innovation and social influence in terms of adoption ( Oliveira et al., 2016). Consequently, it is the country with the most cited articles, with a total of 4056 accumulated citations, accounting for important articles that also study the role of social applications in the attitudes and behaviors of consumers and their incidence the adoption of mobile payments ( Andronie et al., 2021).

India has the second-most cited articles, with 45 publications and 1439 citations; one of these investigations identifies the determinants of satisfaction with the use of mobile payments that could improve the adoption of this ser-vice, highlighting factors such as cost, usefulness, trust, social influence, credibility, information privacy and responsiveness, and how these drive mobile payment service experiences and affect user satisfaction, all based on data taken of the social network Twitter, mentioning that, according to these findings, service providers should try to minimize the cost of transactions, since if addressed correctly, this could have a positive impact during said mobile payments between parties.

Another of the reference countries is Spain, which, although not among the most productive in the scientific field, is in the second position of impact, with a total of 1750 citations, in important articles where it is evidenced by how the age of potential mobile payment users can generate possible moderating effects on the adoption of this system, since the suggested behavior pattern has an adequate adjustment, evidencing that depending on the age of the users, considerable differences are caused in the proposal between ease of use and trust, the latter concerning ease of use and the attitude that each person has towards its use.

### Conceptual components

Following the analysis of the research and academic production on the subject, this section raises the analysis of thematic components with the idea of studying the research evolution around the most frequent key concepts in the literature, such as trend analysis and identification of emerging words of greater relevance to the object of study.

**Figure 6.**
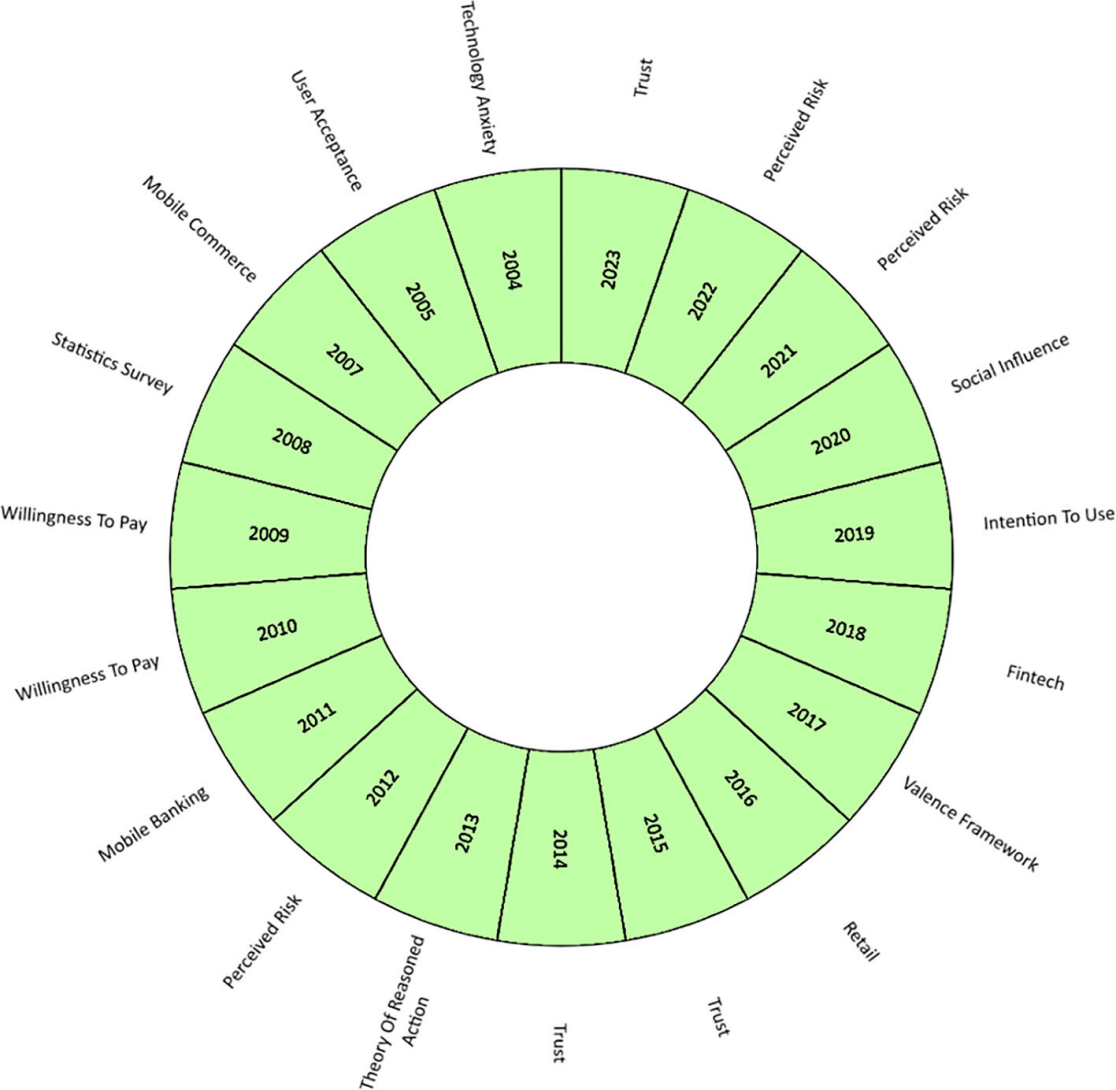
Concept evolution. **Alt text** **Figure 6**. Diagram illustrating the concept evolution of the subject.

Thus, Figure 6 shows an analysis of the evolution of academic production on mobile payment adoption factors, where the main concepts are highlighted by year, including from the year of the first publication on the subject (2004). Hence, in the first place, trust is postulated as the most relevant term and trend for 2023, in addition to being a term that has been repeated in the literature during 2014 and 2015. Trust has been analyzed in the literature as a key factor in consumers conducting business transactions online or participating in e-commerce and internet banking processes. This factor has been defined as the expectation that other users fulfil their promises and obligations in the transactional exchange relationship ( Yang et al., 2023). Confidence has been verified in various studies as the determining factor or predictor of the intention to adopt mobile applications to make online purchases [35], ( Yang et al., 2023).

At the same time, the frequency of perceived risk increased, standing out in the literature during 2012, 2021, and 2022. The publication [36] has argued the importance of analyzing the risk under the psychological behavior of users of virtual and online purchases, who associate this emotion with uncertainty due to the possibility of economic losses in transactions mediated by the internet.

During the last decade, the evolution of mobile payment studies has changed according to technological advances in the economic sectors. In that period, factors such as social influence predominated in the literature, characterized in part by human relations, the internet, social networks and the perception of the use of technology that the social reference group has of the potential use of mobile payments, such as relatives, friends, colleagues ( Wei et al., 2021); fintech, the development of technological applications in finance that has increased access to various banking services in digital media ( Hassan et al., 2019). Technological advances have also been combined with theories that study the behavior of the individual against the intention of use and the theory of reasoned action in making decisions to adopt digital platforms to make payments for products and services, whose application by parts of companies is to attract and retain their customers ( Ariffin & Lim, 2020; F. Liébana-Cabanillas, Molinillo, et al., 2020).

**Figure 7.**
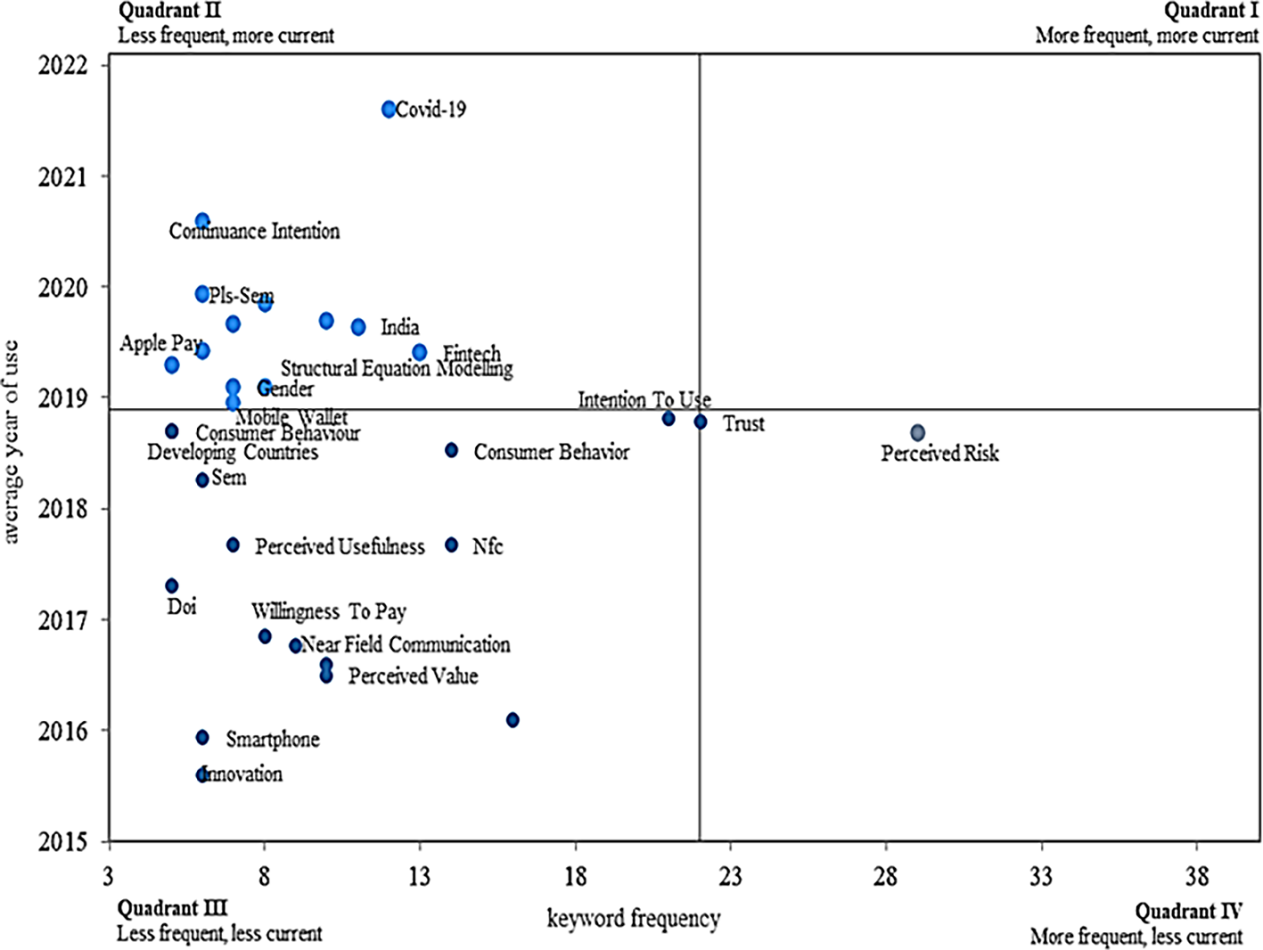
Validity and frequency of keywords. **Alt text** **Figure 7**. Graph representing the validity and frequency of keywords in the dataset.

In terms of the frequency and validity with which the keywords above are applied according to the adoption of mobile payments, as shown in Figure 7, from the graph of a Cartesian plane, four different quadrants are identified, which reflect the concepts with a higher level of appearance and at the same time have decreased in importance today associated with the topic of interest.

Therefore, in quadrant II, one of the less frequent words, but in turn with a high average of validity, is fintech (financial technology), presented in the study on the growth challenge of mobile payment platforms from a business model perspective. In this study, it is recommended that mobile payment providers should adapt to their role within the commercial ecosystem to position themselves, which will depend on the scope and geographic availability that each one has ( Jocevski et al., 2020).

On the other hand, quadrant III contains the least current keywords used with less frequency, such as smartphone, consumer behaviour, perceived usefulness, near field communication, perceived value, mobile commerce, innovation, and willingness to pay.

Finally, quadrant IV, where the least current words that are related to the subject of study but that are used with the greatest frequency are identified, contains the term perceived risk, making an appearance in an article that focuses on the construction of a model that brings together risk into a perceived value from multiple sides, based on the opinions of consumers about the adoption of payments without the need to use credit cards, making use of solely of their smartphone, thus discovering that the perception of risk-value is a remarkable catalyst in this system of interest, taking into account the utilitarian and enjoyment values as the main motivators of the user, in addition to the psychological and privacy risks such as most important convincing elements ( Cocosila & Trabelsi, 2016).

### Theory analysis

This section identifies the main theories that have tried to explain the phenomenon of the adoption and use of mobile payments through multiple models. Table 1 presents 9 typologies of the most common theories in the academic production of the subject studied from 2004 to 2022, showing that over the years, many models have been developed in the various economic sectors. Among them, the following are identified:

**Table 1.** Adoption theories in mobile payments.

Model	Frequency	Authors
TAM extended	23	( Yan et al., 2021); ( Vandana & Mathur, 2022); ( Tounekti et al., 2020); ( Sleiman et al., 2021); ( Sembiring et al., 2022); ( Schmidthuber et al., 2020); ( Quan et al., 2023); ( Qu et al., 2018); ( Peng et al., 2012); ( Lwoga & Lwoga, 2017); ( Lui et al., 2021); ( Muñoz-Leiva et al., 2017); ( F. Liébana-Cabanillas et al., 2015); ( Li et al., 2019); ( Lew et al., 2020); ( Koenig-Lewis et al., 2015); ( Kim & Kim, 2022); ( Ibrahim et al., 1 C.E.); ( Gbongli et al., 2019); ( de Luna et al., 2019); ( Almajali et al., 2022); ( Agárdi & Alt, 2022); ( Josephng et al., 2022)
UTAUT Extended	12	( Wei et al., 2021); ( Singh & Somaiya, 2020); ( Patil et al., 2020); ( Ojo et al., 2022); ( Możdżyński & Cellary, 1 C.E.); ( Malarvizhi et al., 2022); ( Liu et al., 2022); ( Lin et al., 2021); ( Lee et al., 2019); ( Chang et al., 1 C.E.); ( Akanferi et al., 2022); ( Migliore et al., 2022)
UTAUT	3	( Ariffin & Lim, 2020); ( Zhao et al., 2021); ( Hee et al., 2020)
TAM	3	( Nan et al., 2020); ( Hee et al., 2020); ( Zhao et al., 2021)
IRT	2	( Kaur et al., 2020); ( Migliore et al., 2022)
TPB Extended	1	( Belanche et al., 2022)
TPB	1	( Josephng et al., 2022)
ECT	1	( Nan et al., 2020)
Own models	17	

The technology acceptance model (TAM), initially proposed by Davis in ( Davis, 1989), has been taken by researchers as the basic theoretical framework to explore the adoption behavior of mobile payments of users. This theory arises by modifying the theory of reasoned action (TRA) defined in ( Fishbein & Ajzen, 1977), such as social pressure from others on the behavior and decisions of an individual. This modification that determined the TAM model was used to explain to the users their intention to use and adopt new technologies, considering as predictive variables of this phenomenon the ease of use (EOU), the perceived usefulness (PU), and attitude towards use (ATU) ( Al-Husamiyah & Al-Bashayreh, 2022).

At the same time, the TAM model has been modified as it has been implemented in various contexts, justified by the particularities that consumers may have for each technology ( Pliatsikas & Economides, 2022). For example, ( Vandana & Mathur, 2022) presented an extended model that added to the study the variables trust, perceived security, convenience and social influence to predict the use of mobile payments through fintech technologies. In the same way ( Tounekti et al., 2020) raised the factors in the study based on several characteristics of the means of payment: security, perceived ease, privacy, convenience, portability, accuracy, payment term, rate and cost and secure payment methods to determine the variables that consider the consumers to prefer a means of payment.

While these modifications were emerging, some studies addressed the unified theory of acceptance and use of technology (UTAUT) developed by ( Venkatesh et al., 2012), which are analyzed as variables responsible for the behavioral intention to adopt a technological system at the performance expectation, effort expectation, social influence and facilitating conditions. An example of applications is ( Zhao et al., 2021), which proposes a theoretical adoption model integrating the UTAUT and two additional variables: confidence and perceived security; thus, it is evident that all these theories make significant contributions by combining more extensive models with other at-tributes of human behavior, while the findings from Al-saedi et al.’s (2020) research suggest that performance expectancy is the primary determinant of the intention to use a mobile payment system, with social influence, effort expectancy, perceived trust, perceived cost, and self-efficacy following in that order.

That compared to what Lisana (2021) proposed self-efficacy has the largest total effect on behavioral intention, followed in decreasing order of importance by perceived usefulness, perceived ease of use, social influence, trust, network externalities, and uncertainty avoidance.

On the other hand, the innovation resistance theory (IRT) examines behavior based on the reasoning for decision-making related to the use of innovations due to the possible impacts of their applications and changes in consumer belief systems ( Hew et al., 2019). This individual resistance can be relevant in the success or failure of developing new technologies, especially since innovations will continue to make their way into the market. The latter has recently led to the recognition of IRT in the literature as the theoretical basis for adopting mobile payments ( Kaur et al., 2020). Since according to Handarkho and Harjoseputro (2019) consumer innovativeness is the factor that most directly influences the adoption of mobile advertising, followed by willingness to negotiate, perceived convenience and perceived herd behavior.

The work of ( Kaur et al., 2020) proposed a model to evaluate the psychological and functional barriers as variables that explain the adoption of mobile payments, taking into account the impact on consumer behavior for the first time and in the continued use of this technology. In this sense, IRT has also recently been integrated into studies of acceptance of mobile payments, combined with other theories such as the UTAUT, as in ( Migliore et al., 2022), where it was proposed to identify the current psychological and functional barriers, which are key in the intention to use mobile payments between Italy and China.

Another frequent theory in the literature is the theory of planned behavior (TPB), which states that the behaviors of the individual are determined by three key factors: attitude, subjective norms, and behavioral control ( Josephng et al., 2022). The TPB emerges from the TRA of Fishbone and Janzen 1975, which was modified by Ajzen from 1985 to 1991, adding the variable perceived behavioral control, which was relevant when explaining the decision of the users ( Josephng et al., 2022). Thus, this dimension included a general vigilance of the consumer, where perceived behavioral control (PBC) depends on the person’s particular capacities and internal and external limitations.

Additionally, the Expectation Confirmation Theory (ECT) is observed in the literature, which proposes explaining the characteristics of the consumer who decides to repurchase or intends to continue using a digital payment means ( Hadji & Degoulet, 2016). Among these studies ( Nan et al., 2020), combines the ECT model measured mainly by the satisfaction factor with the dimensions of the TAM model theory to explain the rapid spread of social mobile payment.

### Analysis of the main variables

Likewise, reviewing the theories described above allowed us to reveal the most representative variables in the academic production of the subject analyzed from 2004 to 2022, as presented in Table 2, relating the variables to the authors who have addressed them.

**Table 2.** Most important variables of the adoption of mobile payments.

Variable	Frequency
Perceived trust	45
Perceived ease of use	36
Perceived usefulness	35
Perceived risk	31
Perceived social influence	29
Perceived expectation	26
Attitude towards use	14
Facilitating conditions	11
Perceived enjoyment	11
Perceived personal innovation	10
Perceived quality of the system	9
Behavioural intention	9
Perceived convenience	8
Perceived compatibility	7
Perceived value	7
Perceived benefits	5
Perceived cost	5
Gender	5
Use of mobile payments	5

From there, 19 variables are observed, which are defined considering the meanings established by the authors in the development within each model or applied context. First, perceived trust is the most commonly used variable in studies of digital payment adoption, and it lack is one of the main reasons why online payments are not adopted. Thus, trust is defined as the level of risk associated with the use of technological innovations and as when the consumer does not have information or a reliable link before the preliminary encounter with technology ( Talwar et al., 2020).

In the TAM model, perceived ease of use is a variable analyzed in the studies as the clear and understandable interaction between the user and the mobile payment technology tool is understood as an activity that does not require any mental effort on the part of the consumer (F. Liébana-Cabanillas et al., 2015). In this sense, the perceived useful-ness factor is related to the fact that the user’s decision to adopt online payments is determined when he evaluates whether, in these services, he finds a possible beneficial result ( Zhao et al., 2021).

In the evolution of the revised academic production, one of the studies that mostly covers the most common concepts or dimensions to try to predict the adoption of online payments is “Predicting the Intention and Adoption of Near Field Communication Mobile Payment” by ( Malarvizhi et al., 2022). For example, the perceived risk variable was detailed as an additional factor for the extended UTAUT and measured the degree of uncertainty the user has regarding the threats of using technology for the first time. Perceived social influence corresponds to the effect of the opinion of family, friends, relatives, and superiors on consumers and their adoption of mobile payments. Another dimension this study defines is the perceived expectation associated with the user’s expected performance. They will be interested in using online payments if they are considered advantageous and capable of improving their performance in said procedure or activity.

Another important factor that is highlighted in this review is attitude, which, according to what is stated in ( Ariffin & Lim, 2020), has been validated and measured in the theory of planned behavior model to explain the feelings of individuals regarding whether their behavior is favorable or unfavorable to the use of technology. Additionally, according to ( Josephng et al., 2022), attitude can be due to motivations, intensity and negative or positive convictions that influence consumer behavior when making payments online.

Meanwhile, the innovative behavior of the individual has also been studied to predict the intention to use the technologies that allow mobile payments. In this respect, ( Najdawi et al., 2021) defines the perceived personal innovation factor as the level at which the user is considered a technological pioneer and is a determining factor influencing the adoption of electronic payment systems. Other factors arise from the user’s experience with technology; for example, the perceived enjoyment dimension is defined as the satisfaction related to the intention to use any techno-logical product or service ( Sleiman et al., 2021).

Finally, the interest of the subject is highlighted by analyzing factors related to the perception of value generation provided by the use of online payments in such a way that the same variable has taken various names: the perceived value factor has been used to measure the perception of the value of technological innovations in customers based on the risks and benefits generated with their adoption; in relation to perceived convenience, this evaluates the combination of the usefulness of time and place in the use of the means of payment ( Al-Qudah et al., 2022); and finally, the dimension perceived benefits has tried to explain the psychological conditions responsible for the perception of benefit, which determines the use of online payments ( Zhao et al., 2021).

It is important to highlight that, although the “gender” variable is commonly used as demographic information or moderating factor in the instruments, it stands out as one of the most frequently mentioned in the formulation of hypotheses in various studies. For example, Wei et al. (2021) identifies it as crucial in modulating the effects of various factors on mobile payment adoption. Likewise, Liu et al. (2022) examines its impact on consumers’ behavioral intention toward the use of mobile payment apps. These researchers explored whether gender had any effect on performance expectations, ease of use, and social influence with respect to consumers’ behavioral intention toward mobile payment application.

## Discussion

After examining the research and academic production related to mobile payment adoption, key trends around the evolution of studies stand out. The essential theme emerges from literature related to “Theoretical Adoption Models,” where various theories have been proposed to explain the phenomenon of mobile payment adoption and usage. Notably, the Technology Acceptance Model (TAM) and the Unified Theory of Acceptance and Use of Technology (UTAUT) have been highlighted. These models have undergone modifications and adaptations to different contexts, reflecting the complexity of factors influencing user decisions and emphasizing the “Significant Variables in Adoption” of mobile payments.

In this context, one of the highlighted aspects is trust, identified as the preeminent factor and projected as the most prominent trend for 2023. Trust, as per literature, is addressed in mobile payment adoption through the functional reliability of mobile payment systems, general willingness to trust, and cultural background, particularly uncertainty avoidance. These are key factors influencing consumers’ intention to adopt mobile payment ( Xin et al., 2015).

It is mentioned that trust can have a positive effect on mobile payment adoption. When customers perceive mobile payment providers and their applications as reliable, they are more likely to adopt them. Furthermore, trust is linked to changes in customer payment habits ( Hasan, Ashfaq & Shao, 2021), highlighting the need for companies to invest in effective security measures ( Allahham & Ahmad, 2024).

This is due to the concept of “Perceived Risk,” which has experienced a notable increase in frequency, especially in the years 2012, 2021, and 2022. It is argued that the perception of risk is closely linked to emotions and the uncertainty associated with potential economic losses in online transactions. Research emphasizes the importance of analyzing risk from a psychological perspective regarding user behavior in virtual purchases and online transactions, noting that consumers have concerns about the risk of using mobile payments and need assurances that their personal information is secure.

It is suggested that a lower perception of risk and increased security can enhance trust among consumers and their intention to use mobile payments. Additionally, it is noted that, despite potential risks in mobile payments, security measures such as passwords, facial or fingerprint recognition have been implemented to protect users, improving the security of these services ( Hasan et al., 2023). From this, perceived risk is considered a formative construct, proposing it as a second-order factor of seven defined risks: financial risk, performance risk, psychological risk, time risk, security risk, social risk, and privacy risk ( Ramtiyal, Verma & Rathore, 2022).

Moreover, literature focuses on “Social and Technological Influence” in the evolution of mobile payment studies. In the last decade, there has been a shift towards social influence factors, paying special attention to human relationships, social networks, and technology perception. This shift correlates with significant technological advances, such as the growing importance of Fintech, which has expanded access to various banking services in digital environments ( Hassan et al., 2019). Additionally, the combination of technological advances with theories exploring individual behavior and usage intention reveals the interconnection between the technological sphere and social relationships, indicating the need to address these aspects together in future research ( Wei et al., 2021; Ariffin & Lim, 2020).

The Fintech theme is extensively addressed in the study, emphasizing the importance of financial technology adoption. Key drivers of Fintech adoption are mentioned, such as smartphone development, mobile internet technologies, and the convenience of mobile payments. Furthermore, the challenges and opportunities faced by companies are discussed ( Hasan, Ashfaq & Shao, 2021).

The Fintech theme is currently present in the study of the evolution of payment systems and transfer networks, especially in the context of mobile technologies and virtual wallets. It highlights the transformation of digital strategies in the financial landscape by offering innovative electronic deposit solutions, driven by a combination of technological advances, regulatory changes, and simpler organizational structures that enable faster deployment of payment technologies than traditional banks. Analyzing the transactional behavior networks of users is mentioned as providing valuable information about the adoption of new payment technologies and the evolution of mobile payment systems ( León, 2021).

In this way, the current theme, viewed from different perspectives, utilizes cutting-edge technologies to innovate in products and create differentiation in the market, focusing on security, ease of use, and efficiency. It is emphasized that Fintech technologies have the potential to make fundamental financial services more accessible, convenient, and secure. It is projected to enhance emerging economies by addressing challenges present in their adoption ( Patnaik et al., 2023). In this regard, Fintech not only transforms digital strategies but also contributes to making financial services more accessible, convenient, and secure, with the potential to boost developing economies. It presents itself as a complex phenomenon influenced by interconnected factors, where trust, perceived risk, and technological evolution play central roles in shaping the current and future landscape of payment systems.

### Theoretical and practical implications

This research has significant theoretical implications for the field. Firstly, the analysis of the frequency of publications per year indicates an exponential growth in interest and attention dedicated to this topic over time. This increase in academic production reflects the growing importance and relevance of the phenomenon of mobile payment adoption in the current context of digital transformation and changes in consumer behavior.

Secondly, identifying the main theoretical references used by researchers provides a comprehensive understanding of the conceptual frameworks and explanatory models that support the study of mobile payment adoption. The frequent use of models such as UTAUT (Unified Technology Acceptance Model) and TAM (Technology Acceptance Model) highlights the significance of comprehending the psychological and social factors that impact users’ attitudes and behaviors towards these technologies.

Thirdly, analyzing concept evolution helps identify areas of interest and emerging concepts in this field of study. The appearance of terms such as ‘security’, ‘perceived usefulness’, and ‘user experience’ suggests a growing concern for aspects related to trust, effectiveness, and user satisfaction in the context of mobile payments.

Furthermore, the analysis of emerging and growing keywords emphasizes the need to investigate specific aspects, such as the influence of the pandemic on the adoption of mobile payments, the evolution of technologies like QR codes, and the integration of mobile payment systems in different cultural and economic contexts.

Identifying research gaps in the literature provides guidance for future research and the formulation of research agendas. Potential areas of study may include exploring contextual and cultural factors that influence the adoption of mobile payments, designing effective education and communication strategies to promote the acceptance of these technologies, and evaluating the social and economic impacts of their large-scale implementation.

This study lays a strong foundation for comprehending and advancing theory in this field, while identifying research areas that can contribute to the ongoing enhancement of practices and policies related to mobile payment systems.

The findings of this research have several theoretical and practical implications. First, there is no evidence in the literature of bibliometric and systematic reviews focused on strictly analyzing the adoption models and the variables associated with these theories, as is the case of ( Tounekti et al., 2020), which, although recent, does not delve into or compare the variables and theories involved in the acceptance of mobile interfaces to make payments.

It is also clear that as time passes, technological advances undergo improvements that impact the commercial activities of businesses, making it necessary to understand how consumers select their payment methods since this would benefit online retailers, e-commerce sites, virtual payment method providers and web developers. Thus, these results could contribute to guiding policies for emerging companies, managers, government agencies and private service providers interested in mobile payment services.

Consequently, stakeholders with an important role in the electronic commerce system must initially recognize the importance of new payment methods and their operation, as well as the benefits perceived by users, until they focus on taking advantage of the characteristics of the innovation and technology to maintain the quality, reliability and efficiency of the service and at the same time satisfy the physical and mental expectations of the users, thus achieving the acceptance of their products in terms of the design of the system attributes.

The preceding is essential to improve the public experience of the mobile payment user because it would promote a positive social effect on the reputation of technology providers in different situations who are obliged to guarantee the compatibility, efficiency and security of transactions and adapt to the client’s lifestyle.

The results also show that the most popular means to carry out commercial transactions is payment through mobile devices, given the increase in the number of users who use smartphones and because it provides agility and allows more efficient transactions (J. Li et al., 2019), especially under emergency conditions such as the pandemic, a situation in which the stable development of businesses is possible ( Zhao et al., 2021). Therefore, this work expands the literature on the adoption of mobile payments and the interaction between the consumer and innovative technologies, reflecting the motivations of users in the search to accept alternative payment mechanisms. Likewise, this research offers new insights by comparing the different factors and theories that explain the intention to use and adopt online payments.

This study offers a broad and precise overview of the emerging issues and the theoretical evolution related to accepting payments through electronic devices. It provides information that is also relevant to conceptualize this issue better; therefore, it also contributes by guiding thematic lines for future research. In this regard, this study has built a research framework on mobile payment that will serve as a practical guide so that academics and professionals can consult to contextualize themselves and address future studies without problems.

This knowledge framework will allow the establishment of new models based on the variables that are trends and in which they can be derived from the research agenda proposed in this study.

Finally, the value of this document also lies in disseminating knowledge to an important audience that includes professionals and researchers in areas similar to the development of electronic interfaces for payments, which out-lines and identifies future research opportunities.

The research findings emphasize the importance of considering the expectation of effort as a determining factor in the adoption of mobile payments. Studies by Yu et al. (2021) and Fedorko et al. (2021) demonstrate how the perception of difficulty or complexity in using these platforms can significantly influence users’ willingness to adopt them. In this sense, companies and developers should prioritize designing intuitive interfaces and fluid payment processes that minimize user effort. This focus on ease of use not only improves the user experience but can also be a determining factor in the competitiveness and long-term success of mobile payment solutions in an increasingly saturated and demanding market.

Similarly, the importance of clear and informative communication regarding the benefits and ease of mobile payments is highlighted by these findings. Therefore, companies and financial service providers should try to clearly explain how mobile payment platforms can simplify financial transactions and reduce the cognitive and operational burden for users. Proactive and transparent communication can decrease perceived barriers related to effort and build user confidence in the security and effectiveness of mobile payment solutions. This ultimately provides clarity in communication and education about the practical benefits and ease of use of mobile payments, which can be important catalysts for greater consumer adoption and acceptance.

By understanding the underlying theories that inform technology adoption and the factors that influence it, researchers and practitioners can design more effective strategies to encourage acceptance and use of mobile payments. For instance, companies can develop more user-friendly interfaces and intuitive payment processes by identifying key variables that affect the intention to adopt mobile payments, such as effort expectancy and perceived usefulness. This, in turn, can increase user satisfaction and adoption of these technologies. Furthermore, practitioners can direct resources and efforts towards research and development of innovative solutions that address user needs and concerns by recognizing research gaps and emerging areas of development. This strengthens the theoretical and practical foundation in the field of mobile payment adoption.

### Limitations

This study has some limitations. First, for the selection and refinement of the data, the researchers examined the title, abstracts, and content of the article to minimize the presence of irrelevant articles; therefore, high-quality scientific articles that were deficient in choosing relevant keywords could have been excluded. At the same time, for the analysis of this work, the Scopus and Web of Science databases were evaluated and used, but other databases of scientific literature are undoubtedly important sources to capture existing thematic trends, such as Google Scholar, PubMed, IEEE Xplore, ProQuest or EBSCO; therefore, the search should be broadened by including them as well.

From the data analysis, the nature of the bibliometric study methodology represents a limitation. Hence, it is recommended to explore combinations of bibliometric techniques and quantitative and qualitative approaches, as in this study, but this relationship between the findings can be complicated, according to Wallin (2005). In this sense, estimating the number of citations analyses the scientific impact of the field of knowledge, but it may tend to underestimate the most recent articles. In terms of the analysis of countries, an author’s affiliation was considered, but not where the authors are from or their nationalities. Furthermore, only articles in English were included in this study. Despite these limitations, the results presented provide a valuable contribution to the knowledge related to adopting online payments.

It is acknowledged that the use of the PRISMA methodology may not be necessary for this study on mobile payment adoption. However, this may limit the inclusion of valuable insights from relevant studies. Considering the extensive research in this field, a more inclusive approach may be preferable to encompass diverse perspectives and findings. Excluding significant studies could reduce overall understanding of the trends and factors influencing mobile payment adoption.

Finally, it is recognized that the wide range of years covered in this study, from 2004 to 2023, could introduce certain limitations in the ability to accurately capture the most recent and relevant dynamics. For future research, it is suggested that a more specific and narrower period be considered, perhaps limiting the analysis to the last three to five years. This strategy would allow for a more focused assessment of the most current developments and provide a sharper perspective on trends and changes that have occurred in a more recent time frame.

### Research gaps

In continuity with this study, Table 3 presents the gaps and research questions identified from the analysis and review of the scientific publications available in the field of research on the adoption of mobile payments so that other authors can enter and carry out new studies, with the idea of being able to fill these theoretical gaps, to be able to understand and analyze in depth the factors that determine the adoption of these technologies, thus con-tributing to their successful implementation and maximization of benefits for users and organizations.

**Table 3.** Gaps and guiding questions for future research.

Topic	Research gaps	Research questions
Theories of adoption and use of technology	Need to identify contextual factors (social, cultural, economic, demographic, and geographic) that influence the adoption of mobile payments.	How do contextual factors (social, cultural, economic, demographic, and geographic) influence the adoption of mobile payments?
	Lack of empirical studies that integrate multiple theories to explain the adoption of mobile payments.	How can the main adoption theories be integrated to understand the adoption factors of mobile payments comprehensively?
	Lack of studies examining the adoption of mobile payments in emerging country contexts.	How does the adoption of mobile payments vary in the contexts of emerging countries?
	Need to expand research on the factors that affect perceived trust.	What factors determine perceived trust?
	Need to investigate how emotional aspects influence the adoption of mobile payments.	How do emotional aspects influence the adoption of mobile payments?
	Scarcity of studies using psychobehavioural theories to understand the effects of personality on the adoption of mobile payments.	How does personality influence the adoption of mobile payments?
Variables of adoption of smart homes addressed	Need to investigate the effects of previous experience in the adoption of mobile payments	How does previous experience influence the adoption of mobile payments?
	Need to deepen the importance of the current use of mobile payments	How does the adoption of mobile payments influence its current use?
	Lack of research on the role of attitude towards use in adopting mobile payments.	How does attitude towards use influence the adoption of mobile payments?
	Scarcity of studies investigating the influence of perceived privacy on the adoption of mobile payments.	How does perceived privacy influence the adoption of mobile payments?
	Lack of research on how the perception of value and user satisfaction influence the intention of continued use of mobile payments.	How does the perception of value and user satisfaction influence the intention of continued use of mobile payments?
	Scarcity of studies investigating the influence of age and gender on the adoption of mobile payments.	How do age and gender influence the adoption of mobile payments?
	Lack of research on the influence of convenience on the adoption and continued use of mobile payments.	How does convenience influence the adoption and continued use of mobile payments?
	Need to investigate the influence of accessibility on the adoption of mobile payments, including access to mobile devices and internet connectivity.	How does accessibility influence the adoption of mobile payments?
	Lack of research on the relationship between the perception of security and trust in the adoption and continued use of mobile payments.	How does the perception of security influence trust and the adoption and continued use of mobile payments?
	Scarcity of studies investigating the influence of cost and convenience in adopting mobile payments.	How do cost and convenience influence the adoption of mobile payments?
	Lack of research on how adoption variables vary in different cultural contexts.	How do adoption variables vary in different cultural contexts?
	Need to investigate the effects of user experience on the adoption and continued use of mobile payments.	How does the user’s experience influence mobile payment adoption and continued use?
	Lack of research on the factors that influence the intention to continue using mobile payments.	What factors influence the intention to continue using mobile payments?
	Scarcity of studies investigating the relationship between perceived security and the adoption of mobile payments.	How does perceived security influence the adoption of mobile payments?
Growing and emerging themes	Need to investigate the influence of promotions and discounts on the adoption of mobile payments.	How do promotions and discounts influence the adoption of mobile payments?
	Lack of research on the factors that influence the adoption of mobile payments in small and medium businesses.	What factors influence the adoption of mobile payments in small and medium businesses?
	Need to investigate how communication and education can increase the adoption of mobile payments.	How can communication and education increase the adoption of mobile payments?
	Lack of research on the relationship between mobile payment adoption and customer loyalty.	How does the adoption of mobile payments influence customer loyalty?
	Scarcity of studies investigating the adoption and use of mobile payments in the government sphere.	How are mobile payments adopted and used in government?

### Investigative agenda

**Figure 8.**
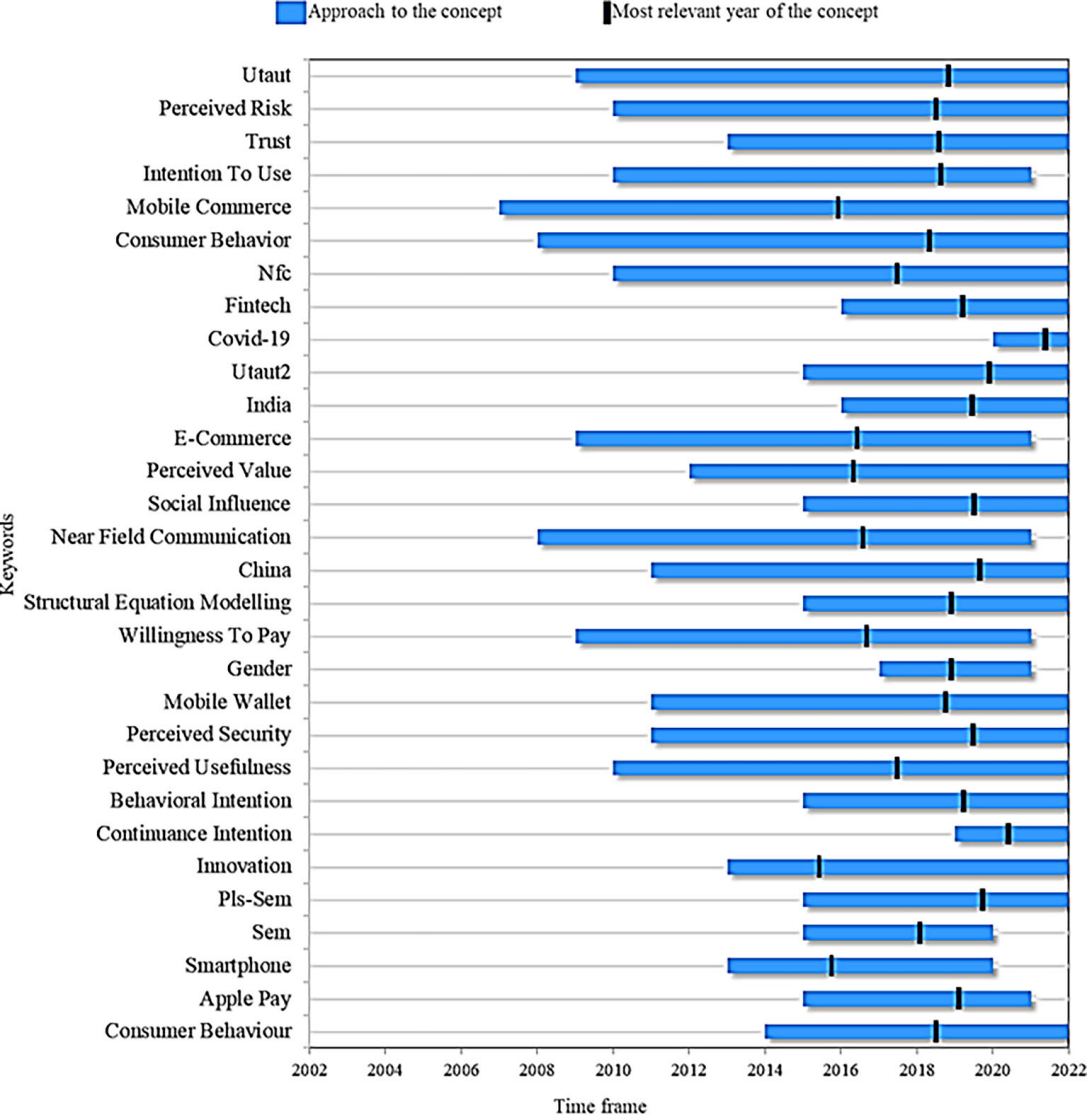
Proposed research agenda. **Alt text** **Figure 8**. Diagram outlining the proposed research agenda.

Finally, Figure 8 presents a proposed research agenda for this systemaztic review with the idea that other researchers can take it as a guide for future scientific studies based on topics considered as trends, emerging and current. First, the 63 articles resulting from applying the PRISMA methodology in the filtering process (inclusion and exclusion criteria) of the records obtained in the search for information were reviewed. From there, this research agenda is proposed after reviewing these documents for the 30 most important terms that the authors have proposed in their studies on the adoption of mobile payments, examining two important points: (1) the time in which the term has been addressed in the literature and (2) the year of greatest relevance in academic production. The latter indicates the period in which the concept had a greater role in academic production, and at the same time, they were studied in the last year.

In this manner, when comparing the concepts of Figure 8, mobile commerce stands out as the term that has been analyzed during a longer window of time, appearing in the scientific literature approximately in 2007 and remaining as a current term in the research to date. Although its most representative year was 2017, it is still positioned among the most relevant keywords, which conceptualizes adopting online payments.

Continuing with the analysis, the keyword that occupies the second place as the term with the highest remaining in the academic production of the subject is consumer behavior. This topic is important to understand, on the one hand, the new challenges, and opportunities that companies face with innovations and, on the other hand, consumer acceptance of technological development, especially to understand the adoption factors for mobile payments. Studies such as ( Zhong & Moon, 2022) highlight the importance of studying the factors associated with user behavior when choosing a virtual payment method. This term then emerged in the literature as of 2009 and is still in force today, with 2019 standing out as the most relevant in the proposed studies.

Among the topics that are presumed to have reached maturity or saturation of information are an intention to use, e-commerce and smartphone. These terms were relevant to building the fundamental conceptual frameworks of the adoption of mobile payments and the theories of acceptance. However, they are no longer valid in the literature, either because of new topics related to technological developments that have updated knowledge or by new needs that the consumer is acquiring.

For example, Figure 8 shows fintech as a current topic, which has appeared in the academic context since 2016 but mainly reflects how technological advances and digitization have disrupted the financial services industry. In this regard, new studies should address variables related to technological factors, internet connectivity and innovative interface design from the business point of view and the relationship of these with online payment service providers to offer a better understanding of their adoption.

In terms of the theories that explain the adoption of online payments, Figure 8 indicates that the UTAUT and extended UTAUT models and the variables innovation, social influence, perceived safety, perceived risk, trust, and perceived usefulness are currently in force and have been the object of study for many years in the literature, which indicates that they will continue to be a fundamental part of the subject in future studies.

On the other hand, when analyzing the terms that are considered recent in the literature and that appear as current trends, from which future research may be deduced, we find continuance intention, appearing in the literature since 2019 and in force this year. This concept has been changing the focus of the studies on using virtual payments for the first time towards their continued use. That is, it works on issues in a transversal way related to user satisfaction and experience, seeking to increase motivation in them, towards loyalty to the services and against the intention of continuing to use the mobile payment system ( Duy Phuong et al., 2020).

This perspective has been developed by several researchers, which justifies a more detailed analysis of how consumers’ reactions affect when the company, through its strategies in the sale of services and products, implements variables such as discounts on the invoice, return payment of cash and gifts.

Another novel approach is the appearance of COVID-19 in 2020 and its year of greatest relevance in the literature in 2021. This is understandable due to the significant impact on markets and online payment systems that the pandemic had on consumers and companies that adopted them to survive in the industry ( Cao, 2021). At the same time, this topic, which is expected to continue to rise in academic production, invites business studies through two important variables: the ability to use mobile technologies and the benefits of mobile payments. The preceding also reveals that companies could direct their efforts to promote services to users with experience in mobile devices due to the rise of these due to the pandemic.

Faced with the methodologies implemented in the studies analyzed, Figure 8 also shows that since 2016, methods such as PLS-SEM and structural equation modelling (SEM) continue to be present today as a valuable multivariate analysis technique to test structural models on the adoption of mobile payments.

Likewise, the case studies using the concepts of India and China are reported as methods that are still useful to enrich the different contexts in which the adoption of mobile payments is implemented, in addition to being cases that stood out as the greatest relevance in the literature for 2019.

It is noteworthy that the detailed review that was carried out in this study also allowed us to recognize some fields that, although not listed in the graph shown, are expected to be a trend in the short term, as they are derived from variables already analyzed in the theories of adoption of mobile payments, such as the performance and user expectations (efficiency, speed, flexibility and security) in transactions. These fields are biometric authentication, payment flexibility, social purchases (on social media), and contactless payments.

## Conclusions

This study identified the research trends associated with adopting mobile payments to guide an agenda for future studies. The analysis allowed a comprehensive review through bibliometric indicators and a systematic literature review of 63 documents. The results allow us to conclude that the publications around the adoption of mobile payments follow an upwards trajectory where researchers have addressed many theories to explain this phenomenon. Among them, the main trends stand out: the technological acceptance model (TAM) and the unified theory of technology acceptance and use (UTAUT), which in the literature have been modified, including new factors to be worked on extensively in various contexts. In relation to this, the variables innovation, social influence, perceived safety, perceived risk, trust, and perceived usefulness are postulated as research trends that predict the behavior of intention to use mobile payment methods by potential users. In addition to the above, this study identified among the research trends the transition towards an approach for the acceptance of these technologies, such as the continuance intention variable, based on the intention of continued use of mobile services for payment and not on the intention to use technology for the first time, as it had been studied during the first decade of thematic evolution.

In future studies, the research agenda suggests considering some guidelines, such as biometric authentication, payment flexibility, social purchases (in social networks) and contactless payments, and at the same time, from the business point of view and the relationship between these with online payment service providers, suggests developing studies on technological factors, internet connectivity and innovative interface design.

## Ethics and consent

Ethical approval and consent were not required.

## Data Availability

Underlying data: No data are associated with this article. Zenodo: Research trends in mobile payment adoption: Research trends and agenda, https://doi.org/10.5281/zenodo.14426875 ( Valencia-Arias et al., 2024). The project contains the following data
• Database • Flowchart diagram • PRISMA checklist The data availability statement for this study has been duly registered and archived in the Zenodo open data repository, which is recognized for its commitment to the accessibility and preservation of scientific data. The data and materials supported by this study are publicly available under a Creative Commons Attribution 4.0 International (CC BY 4.0) license.
