# Supplementary material for: Research trends in mobile payment adoption: Research trends and agenda
Source: F1000Res. 2025 Aug 20;14:358. Originally published 2025 Mar 31. [Version 2] doi: 10.12688/f1000research.159551.2 (PMC12405856; doi:10.12688/f1000research.159551.2)
Supplement: Peer review history

# Reviewer report: Reviewer response for version 2

**DOI:** 10.5256/f1000research.185656.r407431

**Published:** 2025-09-05

**Authors:** Mohammad Rakibul Islam Bhuiyan, Lisa Mani

**Affiliations:** 1 Begum Rokeya University, Rangpur, Bangladesh; 2 Finance and Banking, Shahjalal University of Science and Technology School of Management and Business Administration (Ringgold ID: 309531), Sylhet, Sylhet, Bangladesh

Thank you for revision. My decision is to accept this paper without any reservation.

Are the rationale for, and objectives of, the Systematic Review clearly stated?

No

Is the statistical analysis and its interpretation appropriate?

Yes

If this is a Living Systematic Review, is the ‘living’ method appropriate and is the search schedule clearly defined and justified? (‘Living Systematic Review’ or a variation of this term should be included in the title.)

No

Are sufficient details of the methods and analysis provided to allow replication by others?

No

Are the conclusions drawn adequately supported by the results presented in the review?

No

Reviewer Expertise:

Economics of Information Systems, Digital Payment Technology, Advanced Manufacturing, Information Privacy & Security, Industry 4.0

We confirm that we have read this submission and believe that we have an appropriate level of expertise to confirm that it is of an acceptable scientific standard.

# Reviewer report: Reviewer response for version 2

**DOI:** 10.5256/f1000research.185656.r407430

**Published:** 2025-09-02

**Authors:** Ni Made Estiyanti

**Affiliations:** 1 Primakara University, Bali, Indonesia

Reviewer Report: Response to Author Revisions

 I appreciate the authors’ careful and thorough response to my initial review comments. The revised manuscript has been significantly improved in terms of methodological clarity, academic rigor, and analytical transparency.

 The authors have successfully addressed all the key points I raised:

 1. Database Search Timeframe: The inclusion of the exact dates for the database searches in Scopus and Web of Science enhances the reproducibility of the study and strengthens the methodological foundation. This is a valuable addition.

 2. Inclusion of Scholarly References: The revised introduction now includes updated and relevant citations that support the key claims. This greatly improves the scientific credibility of the paper and grounds the discussion in existing literature.

 3. Clarity of Analytical Tools: The clarification regarding the use of Microsoft Excel, VOSviewer, and CiteSpace — including their specific roles in the analysis (e.g., burst detection in Figure 8 using CiteSpace) — has significantly improved the transparency and rigor of the methodological section. I appreciate the detailed mapping provided.

 4. PRISMA Flow Diagram: The updated PRISMA diagram, with clear explanations for “report not retrievable” and “record excluded,” now provides a more transparent and comprehensive view of the screening process. This strengthens the trustworthiness of the literature selection.

 5.  Practical Case Studies: The inclusion of real-world examples of successful mobile payment systems adds valuable practical context to the theoretical discussion. This enhancement bridges the gap between research and application, which is essential for readers in both academic and industry settings.

 Overall, the authors have made substantial and meaningful revisions that have elevated the quality of the manuscript. The paper now offers a well-structured, methodologically sound, and insightful contribution to the field of mobile payment adoption research.

 ✅ I am pleased to recommend this manuscript for acceptance in its current form.

Are the rationale for, and objectives of, the Systematic Review clearly stated?

Yes

Is the statistical analysis and its interpretation appropriate?

Yes

If this is a Living Systematic Review, is the ‘living’ method appropriate and is the search schedule clearly defined and justified? (‘Living Systematic Review’ or a variation of this term should be included in the title.)

No

Are sufficient details of the methods and analysis provided to allow replication by others?

Partly

Are the conclusions drawn adequately supported by the results presented in the review?

Yes

Reviewer Expertise:

Financial Technology Adoption, Entrepreneurial Finance, Start-up Entrepreneurship, Sustainability.

I confirm that I have read this submission and believe that I have an appropriate level of expertise to confirm that it is of an acceptable scientific standard.

# Reviewer report: Reviewer response for version 1

**DOI:** 10.5256/f1000research.175296.r394216

**Published:** 2025-07-28

**Authors:** Wahyu Hidayat

**Affiliations:** 1 Universitas Padjadjaran, Bandung, West Java, Indonesia

1. The authors did not conduct a bias test to assess each selected article based on the PRISMA flow, so the method used to select the quality of the chosen journals cannot be justified.

 2. As LSR cannot be fully conducted because there is no bias test method that has not been performed for the selected articles, but if there is already a bias test method for the articles, then LSR can be conducted.

Are the rationale for, and objectives of, the Systematic Review clearly stated?

Yes

Is the statistical analysis and its interpretation appropriate?

Yes

If this is a Living Systematic Review, is the ‘living’ method appropriate and is the search schedule clearly defined and justified? (‘Living Systematic Review’ or a variation of this term should be included in the title.)

Partly

Are sufficient details of the methods and analysis provided to allow replication by others?

Partly

Are the conclusions drawn adequately supported by the results presented in the review?

Yes

Reviewer Expertise:

Herbal medicine, oral medicine, infection and immunogenetics

I confirm that I have read this submission and believe that I have an appropriate level of expertise to confirm that it is of an acceptable scientific standard, however I have significant reservations, as outlined above.

# Response

**Published:** 2025-07-30

**Authors:** JHOANY ALEJANDRO VALENCIA ARIAS

We would like to thank the reviewer for their valuable observations, which have allowed us to strengthen the methodological transparency of our study.

 Regarding the first comment, we understand the concern about the justification of the quality assessment method applied to the selected studies. We clarify that the PRISMA flow diagram was used strictly to document and guide the selection process of studies, but not as the instrument for assessing methodological quality. To evaluate the risk of bias and overall methodological rigor of each included article, we employed the AMSTAR-2 framework, a widely recognized tool designed specifically for the critical appraisal of systematic reviews. As shown in Table 1, this assessment included dimensions such as the justification of inclusion criteria, the transparency and scope of the search strategy, the identification of potential sources of bias, and the evaluation of heterogeneity and publication bias. Each article was independently rated across these dimensions to provide a comprehensive quality profile.

 In response to the second comment, we confirm that a bias assessment method was indeed applied, using the AMSTAR-2 criteria to enable a structured and replicable approach to the evaluation of risk of bias. This allowed us to classify the methodological quality of the 63 studies and to identify both the strengths and limitations of the current literature on mobile payment adoption. We therefore consider that the study meets the minimum criteria required to be framed as a Living Systematic Review (LSR), as it includes a robust and transparent quality appraisal protocol that can be updated and expanded over time.

 We appreciate the opportunity to clarify these aspects and have revised the manuscript to make this information more explicit.

# Reviewer report: Reviewer response for version 1

**DOI:** 10.5256/f1000research.175296.r391989

**Published:** 2025-07-23

**Authors:** Ni Made Estiyanti

**Affiliations:** 1 Primakara University, Bali, Indonesia

This article explores the integration of mobile payments into the realm of research, with the objective of elucidating the technological, socio-economic, and cultural factors that influence user experience and satisfaction, thereby attracting a greater number of users. A systematic literature review based on the PRISMA 2020 framework was conducted to select 63 documents from Scopus and WoS. This review explored research trends in mobile payment adoption. The most frequently utilized models for this analysis are the extended Technology Acceptance Model (TAM) and the extended Unified Theory of Acceptance of Technology (UTAUT). The most salient variables in this study are social influence, perceived security, perceived risk, trust, and perceived benefits. Consequently, it is recommended that future research agendas prioritize subjects such as biometric authentication, payment flexibility, and contactless payments. From a business perspective, there is an emphasis on the development of more intuitive user interfaces.

 This article provides a valuable roadmap for future research, particularly in identifying underexplored variables. The topic of this research is highly relevant and timely, particularly in light of the rapid advancements in digital finance and payment systems. The article is well-structured and clearly presented, contributing to its overall readability and coherence. The methodology employed is appropriate and provides meaningful insights for both theoretical development and practical application in the field.

 Nevertheless, with minor revisions,  there are several areas that require further clarification and refinement in order to enhance the clarity, accuracy, and academic rigor of the paper. Below are some key points identified in the article that could benefit from improvement:

 1) Inclusion of Database Search Timeframe

 The article does not specify the timeframe during which the database searches were conducted in Scopus and Web of Science (WoS). This information is crucial, as the number of publications indexed in these databases evolves continuously, potentially influencing the comprehensiveness and accuracy of the literature analysis. To ensure consistency and reproducibility, it is recommended that future studies clearly report the exact dates of database searches and conduct them simultaneously in both Scopus and WoS to minimize discrepancies and maintain methodological rigor.

 2) Inclusion of References as Scientific Evidence

 Several statements presented in the introduction section appear to reflect opinions or conclusions that are not supported by appropriate scholarly references. It is strongly recommended to ensure that all such assertions be accompanied by relevant citations from credible academic sources. Incorporating references will strengthen the validity and credibility of the claims made, grounding them in existing scientific literature and enhancing the overall academic rigor of the paper.

 3) Clarity Regarding the Use of Analytical Tools

 This article does mention the use of Microsoft Excel and VOSViewer; however, the nature of the analysis and the specific analytical methods used remain unclear. For example, in Figure 7, what analytical approach was used, and what tools were utilized? Furthermore, Figure 8 suggests that the visualization is the result of burst analysis using the Scitespace tool. This statement is speculative, as the source material does not explicitly address this point. Clear explanations and elaborations are essential. To enhance methodological transparency and reproducibility, this study should provide a clear mapping between the tools used and the specific analyses conducted.

 4) Clarify the PRISMA Flow Diagram

 The stages in the PRISMA method have already been explained in the section on inclusion and exclusion criteria. However, it is still unclear to me what the criteria are for “report not retrievable = 305” and also for “record excluded = 167.” It would be better to present the criteria in the PRISMA flow diagram.

 5) Discussion on Limited Practical Case Studies

 The number of practical case studies employed to illustrate theoretical insights is limited. It is imperative to provide concrete examples of successful mobile payment systems in diverse contexts.

Are the rationale for, and objectives of, the Systematic Review clearly stated?

Yes

Is the statistical analysis and its interpretation appropriate?

Yes

If this is a Living Systematic Review, is the ‘living’ method appropriate and is the search schedule clearly defined and justified? (‘Living Systematic Review’ or a variation of this term should be included in the title.)

No

Are sufficient details of the methods and analysis provided to allow replication by others?

Partly

Are the conclusions drawn adequately supported by the results presented in the review?

Yes

Reviewer Expertise:

Financial Technology Adoption, Entrepreneurial Finance, Start-up Entrepreneurship, Sustainability.

I confirm that I have read this submission and believe that I have an appropriate level of expertise to confirm that it is of an acceptable scientific standard, however I have significant reservations, as outlined above.

# Response

**Published:** 2025-07-25

**Authors:** JHOANY ALEJANDRO VALENCIA ARIAS

We thank the reviewer and the editorial team for their invaluable contributions to the rigor of the article. Below, we provide a point-by-point response:

**Comment**: 1) Inclusion of Database Search Timeframe The article does not specify the timeframe during which the database searches were conducted in Scopus and Web of Science (WoS). This information is crucial, as the number of publications indexed in these databases evolves continuously, potentially influencing the comprehensiveness and accuracy of the literature analysis. To ensure consistency and reproducibility, it is recommended that future studies clearly report the exact dates of database searches and conduct them simultaneously in both Scopus and WoS to minimize discrepancies and maintain methodological rigor.

**Response**: The search date was explicitly added.

**Comment**: 2) Inclusion of References as Scientific Evidence Several statements presented in the introduction section appear to reflect opinions or conclusions that are not supported by appropriate scholarly references. It is strongly recommended to ensure that all such assertions be accompanied by relevant citations from credible academic sources. Incorporating references will strengthen the validity and credibility of the claims made, grounding them in existing scientific literature and enhancing the overall academic rigor of the paper.

**Response**: The introduction was thoroughly reviewed, and updated references were added.

**Comment**: 3) Clarity Regarding the Use of Analytical Tools This article does mention the use of Microsoft Excel and VOSViewer; however, the nature of the analysis and the specific analytical methods used remain unclear. For example, in Figure 7, what analytical approach was used, and what tools were utilized? Furthermore, Figure 8 suggests that the visualization is the result of burst analysis using the Scitespace tool. This statement is speculative, as the source material does not explicitly address this point. Clear explanations and elaborations are essential. To enhance methodological transparency and reproducibility, this study should provide a clear mapping between the tools used and the specific analyses conducted.

**Response**: The text already specified what type of analysis was performed with each tool, and the information represented in the figures was clarified.

**Comment**: 4) Clarify the PRISMA Flow Diagram The stages in the PRISMA method have already been explained in the section on inclusion and exclusion criteria. However, it is still unclear to me what the criteria are for “report not retrievable = 305” and also for “record excluded = 167.” It would be better to present the criteria in the PRISMA flow diagram.

**Response**: Specific exclusion criteria were added as an explanatory note within the PRISMA diagram for greater clarity.

**Comment**: 5) Discussion on Limited Practical Case Studies The number of practical case studies employed to illustrate theoretical insights is limited. It is imperative to provide concrete examples of successful mobile payment systems in diverse contexts.

**Response**: Two real-life cases of successful mobile payment systems in diverse contexts were incorporated, strengthening the theoretical discussion.

# Reviewer report: Reviewer response for version 1

**DOI:** 10.5256/f1000research.175296.r394215

**Published:** 2025-06-30

**Authors:** Mohammad Rakibul Islam Bhuiyan

**Affiliations:** 1 Begum Rokeya University, Rangpur, Bangladesh

This manuscript offers a valuable bibliometric and systematic review of mobile payment adoption literature through a PRISMA-guided approach, identifying key trends, theories (e.g., UTAUT, TAM), and future research directions. The structure is clear and the topic is timely given the rapid expansion of digital finance.

 However, several critical improvements are needed to strengthen academic rigor, enhance clarity, and update the manuscript’s scholarly relevance.

**Major Comments and Suggestions**

 1. **Update Literature Coverage**

**Issue**: Although the scope spans from 2004 to 2023, many of the included references and patterns emphasize publications from 2016–2021. Yet new developments—like biometric authentication and Web3 applications—have emerged strongly in 2024–2025.

**Recommendation**:

- Update the literature search to **extend through late 2024**, capturing key recent studies (e.g., advances in contactless payments, super-apps in emerging markets).
- Ensure the **final selection reflects the most current studies**, possibly adjusting inclusion criteria to i) recent top-tier journal papers or ii) novel technologies in mobile finance.

2. **Clarify Methodology and PRISMA Flow**

**Issue**: The PRISMA flow is mentioned but specific numbers (e.g., initial hits, duplicates removed, exclusions) are unclear.

**Recommendation**:

- Present a **PRISMA flowchart** in the main text or supplementary materials with explicit counts for each filtering stage.
- Provide criteria for scoring and removing duplicates or irrelevant articles to increase transparency.

3. **Refine Coding and Thematic Analysis**

**Issue**: While TAM/UTAUT usage is highlighted, thematic coding methodology (category definitions, inter-rater reliability) is not clearly described.

**Recommendation**:

- Explain the **coding process**: how themes/categories were defined, who conducted the coding, and whether reliability was assessed (e.g., Cohen’s kappa).
- Discuss how qualitative interpretation mapped keywords to theoretical models like trust, social influence, or effort expectancy.

4. **Enhance Visualizations and Narrative Flow**

**Issue**: Figures such as keyword evolution (Fig. 7) and thematic agenda (Fig. 8) contain many items with unclear visual differentiation.

**Recommendation**:

- Improve figure clarity through better **color schemes**, simplified legends, and highlighted trends.
- In the text, explicitly **interpret each figure**, guiding the reader through the most salient keywords and emerging themes rather than just referencing them.

5. **Balance Research Gaps and Practical Relevance**

**Issue**: Table 3 and the agenda are comprehensive but lack prioritization and alignment with practice.

**Recommendation**:

- Distinguish **high-priority gaps** (e.g., adoption in the context of financial inclusion, data privacy implications, regulatory frameworks) vs. exploratory ones.
- Connect these gaps to **real-world implications**—for policymakers, mobile platform designers, or regulators in digital finance.

6. **Strengthen Limitations and Future Work**

**Issue**: The limitations of the bibliometric method are briefly noted, but there is no discussion of possible biases (e.g., English-language only, citation lag).

**Recommendation**:

- Expand limitations to cover language bias, bibliometric time-lag skew, and potential database (Scopus/WoS) coverage issues.
- Suggest follow-up studies combining **qualitative interviews**, thematic deep-dives, or emerging market case studies to triangulate findings.

**Minor Suggestions**

- Provide **descriptive table captions**, especially where acronyms appear (e.g., clarify TAM, UTAUT).
- Check consistency in referencing formatting and ensure DOI inclusion throughout.
- Consider adding an **acronym glossary** for ease of reading.
- Note any funding or open data sources for replication transparency.

**Concluding Remarks**

 This manuscript addresses an important and evolving research area. With major revisions focused on updating literature, clarifying methods, refining narrative clarity, and strengthening the connection to practice, it could meaningfully advance research in mobile payment adoption which is acceptable with major revisions.

Are the rationale for, and objectives of, the Systematic Review clearly stated?

No

Is the statistical analysis and its interpretation appropriate?

Yes

If this is a Living Systematic Review, is the ‘living’ method appropriate and is the search schedule clearly defined and justified? (‘Living Systematic Review’ or a variation of this term should be included in the title.)

No

Are sufficient details of the methods and analysis provided to allow replication by others?

No

Are the conclusions drawn adequately supported by the results presented in the review?

No

Reviewer Expertise:

Economics of Information Systems, Digital Payment Technology, Advanced Manufacturing, Information Privacy & Security, Industry 4.0

I confirm that I have read this submission and believe that I have an appropriate level of expertise to confirm that it is of an acceptable scientific standard, however I have significant reservations, as outlined above.
